# Hsa-miR-217 Inhibits the Proliferation, Migration, and Invasion in Non-small Cell Lung Cancer Cells Via Targeting SIRT1 and P53/KAI1 Signaling

**DOI:** 10.4274/balkanmedj.galenos.2020.2019.9.91

**Published:** 2020-06-01

**Authors:** Wenxia Jiang, Likun Hou, Juan Wei, Yifeng Du, Yan Zhao, Xue Deng, Xiangdong Lin

**Affiliations:** 1 Department of Pathology and Pathophysiology, Tongji University School of Medicine, Shanghai, China; 2 Experimental Centre of Medicine and Life Science, Tongji University, Shanghai, China; 3 Department of Pathology, Shanghai Pulmonary Hospital, Tongji University, Shanghai, China; 4 Tongji University School of Medicine, Shanghai, China

**Keywords:** Brain metastasis, hsa-miRNA-217, lung cancer, PC-14/B cells, sirtuin 1

## Abstract

**Background:**

Brain metastasis is a major cause of cancer death in patients with lung cancer. Sirtuin 1 and hsa-miR-217 have been identified to mediate the development of non-small cell lung cancer.

**Aims:**

To investigate the roles of hsa-miR-217, its target sirtuin 1, and the P53/KAI1 axis in the brain metastasis from non-small cell lung cancer.

**Study Design:**

Cell culture study.

**Methods:**

Human pulmonary adenocarcinoma brain metastasis cell line PC-14/B were incubated and treated with constructed lentiviral plasmids expressing miR-217 and/or sirtuin 1. BEAS-2B cell line was used as a control. The targeted regulation of miR-217 to sirtuin 1was examined using a dual-luciferase reporter assay. Cell proliferation, migration, invasion, and related protein expression were detected to examine the effect of the miR-217/sirtuin 1 expression on metastasis.

**Results:**

PC-14/B cells expressed higher sirtuin 1 and lower P53 and KAI1 compared with BEAS-2B control cells (p<0.05). Sirtuin 1 was a direct target of miR-217. MiR-217 expression suppressed PC-14/B cell invasion (p=0.004), migration (p=0.001), and proliferation (p<0.05), whereas sirtuin 1 overexpression reversed all processes. sirtuin 1 expression inhibited P53, KAI1/CD82, matrix metalloproteinase-9, and β-catenin but upregulated E-cadherin protein. MiR-217 overexpression induced reverse changes.

**Conclusion:**

Hsa-miR-217 and its target sirtuin 1 acted as metastasis suppressor and promoter gene in non-small cell lung cancer, respectively. The hsa-miR-217/sirtuin 1/P53/KAI1 metastasis regulatory pathway showed novel and crucial roles in brain metastasis from non-small cell lung cancer. This axis might be a potential target for the treatment of brain metastasis of lung cancer.

Brain metastasis is a complication found in about 20-40% of the patients suffering from non-small cell lung cancer (NSCLC) (1). Although surgical therapy, radiotherapy, and novel systemic therapy have made strides in the treatment of brain metastasis from lung cancer in the last two decades, the survival rate remains low, with a typical lifetime of only months (2).

Studies have demonstrated that prophylactic cranial irradiation could decrease the recurrent risk of brain metastasis and intracranial tumor in patients suffering from NSCLC, (3) but its efficacy on improving survival outcomes in NSCLC patients with brain metastasis remains unknown. Whole-brain radiotherapy (WBRT) is the standard treatment of brain metastasis from cancers, including NSCLC. However, WBRT alone has limited survival benefits in patients. The combination of surgery and WBRT extends the survival period of independent treatment and reduces the mortality associated with the nervous system and local recurrence (4). However, this combination reduces health-related living quality (4).

Compared with stereotactic radiosurgery or epidermal growth factor receptor (EGFR) tyrosine kinase inhibitors alone, WBRT supplementation is beneficial for NSCLC patients with two to four brain metastases, including controlling cognitive progression and intracranial tumor (5,6,7). The combination of chemotherapy and WBRT not only increases the response rate and controls brain metastasis but also increases toxic and side effects and did not significantly benefit survival (5,6,7,8). Targeted therapy is a research hotspot in gene therapy of tumors. The identification of new key genes with the potential of inhibiting brain metastasis from lung cancer is indispensable to the development of targeted drugs and precise treatment.

MicroRNAs (miRNAs) and their targets play important roles in the metastasis of cancers. Hsa-miR-217 showed various roles in tumorigenesis and drug development and resistance (9,10,11). Hsa-miR-217 inhibits laryngeal cancer metastasis by suppressing the expression of its targets, including astrocyte elevated gene-1 and programmed death-ligand 1 (11). The theoretical target gene of hsa-miR-217, sirtuin 1 (SIRT1), was highly expressed in the brain metastasis tissues of NSCLC compared with NSCLC tissues (12). Our primary experiments found that SIRT1 had a high expression level in the NSCLC brain metastatic cells compared with that in normal cells. We thus assumed that hsa-miR-217 might play an important role in brain metastasis from NSCLC via targeting SIRT1.

SIRT1-mediated P53 signaling has been validated in various cells (13,14). SIRT1 is a nicotinamide adenine dinucleotide (NAD)-dependent deacetylase, which deacetylates and inhibits its physiological substrate P53 (13). The SIRT1-P53 signaling pathway plays important roles in the metastatic progression of cancers, including prostate cancer (15) and esophageal squamous cancer (16). In addition, a target of P53, the metastasis suppressor gene KAI1/CD82, showed therapeutic potential in NSCLC (17). However, there was no direct report showing the association between hsa-miR-217/SIRT1/P53/KAI1 pathway and brain metastasis from NSCLC.

We performed this study to investigate the roles of hsa-miR-217 and its target gene SIRT1 in the brain metastasis from NSCLC. The cell proliferation, migration, and invasion of PC-14/B cells transfected with hsa-miR-217 and SIRT1 expressing plasmids were detected to evaluate the effect of the hsa-miR-217/SIRT1/P53/KAI1 pathway on cell metastasis. This study would provide a novel insight into the mechanism of the hsa-miR-217/SIRT1/P53/KAI1 pathway-mediated brain metastasis from NSCLC.

## MATERIALS AND METHODS

### Cell culture

The human pulmonary adenocarcinoma brain metastasis cell line PC-14/B was obtained from Shanghai Maisha Biotechnology Co., Ltd (Shanghai, China). The human bronchial epithelial cell line BEAS-2B was a gift from the Department of Physiology, the Second Military Medical University, Shanghai, China. The 293 T-cell line was purchased from the cell bank of the Chinese Academy of Sciences (Beijing, China). PC-14/B and BEAS-2B cells were cultured in RPMI160 medium (Invitrogen, Shanghai, China) supplemented with 10% fetal bovine serum (FBS), and 293 T-cells were cultured in Dulbecco’s modified Eagle medium (DMEM; Invitrogen) plus 10% FBS. All cells were maintained at 37°C in 5% CO_2_.

### Plasmids and vectors

Human genomic DNA was extracted from PC-14/B cells and used for amplification of the precursor sequence of has-miR-217. The polymerase chain reaction (PCR) product was inserted into a linear pCDH-EF1-GFP vector (System Biosciences, Mountain View, CA, USA; pcDH-miR-217) through double enzyme digestion (EcoRI and BamHI) and was then transformed into Top10 competent cells (Takara, Tokyo, Japan). The coding sequence of SIRT1 was amplified and cloned into the pcDH-CMV lentiviral expressing vector (Promega, Madison, WI, USA; pcDH-SIRT1) through double enzyme digestion (HindIII and BamHI) methods. The constructed vectors were validated by DNA sequencing. The mimics, inhibitor, and negative control of hsa-miR-217 were chemically synthesized by Shanghai Sangon (Shanghai, China).

### Preparation of recombinant lentivirus

Twenty-four hours before transfection, 293 T-cells were cotransfected with pcDH-miR-217 (2 μg) or pcDH-SIRT1 vector (2 μg) and 10 μg pPACK Packaging Plasmid Mix (System Biosciences) using Lipofectamine 2000 (Invitrogen). Cells were then incubated in the DMEM with 1% FBS for 48 h, followed by harvest, centrifugation (5000 × g at 4°C for 5 min), and filter. The titer of packaged lentiviruses was determined using gradient dilution.

### Overexpressing hsa-miRNA-217 and SIRT1 in PC-14/B cells through a lentiviral approach

The suspension of PC-14/B cells (logarithmic phase) was prepared using trypsin digestion (Promega), and the density of viable cells was determined. Cells were collected, resuspended, and then seeded into six-well plates with a density of 2×10^6^ cells/well. Cells were cultured in RPMI1640 medium overnight at 37°C in 5% CO_2_. Then, the medium was replaced with fresh medium supplemented with Lv-miRNA-217 and/or Lv-SIRT1 vectors with a multiplicity of infection (MOI) of 10. The infection efficiency and the hsa-miR-217 and SIRT1 contents were determined at 72 h post-infection.

### Luciferase reporter assay

TargetScan was used to predict the miRNA-mRNA target pair. The total RNA was extracted from PC-14/B cells and reversely transcribed into cDNA samples. The luciferase reporter plasmids were constructed by cloning the wild and mutant 3’-UTR of homo sapiens SIRT1 (NM_012238.4) gene into the pGL3-promoter vector (Promega; pGL-WT-SIRT1 or pGL-MT-SIRT1) using Xba I digestion. Hsa-miR-217 mimics, inhibitor, and NC were separately transfected into 293 T-cells together with pGL-WT-SIRT1 or pGL-MT-SIRT1 vectors using Lipofectamine 2000 (Invitrogen), according to the manufacturer’s instructions. After 48 h, 293 T-cells were harvested, and luciferase assays were conducted using a dual-luciferase reporter assay system (Promega).

### Cell proliferation examination

Cell viability was detected using the Cell Counting Kit-8 (CCK-8) assay (Dojindo, Kumamoto, Japan). At 72 h post-infection, a total of 1×10^3^ PC-14/B cells/well (uninfected or infected) were seeded into 96-well plates and incubated for 12, 24, 48, and 72 h. Cell viability assay was performed using a 10 μL/well CCK-8 solution following the manufacturer›s protocol. A microplate reader (MultiSkan Spectrum Thermo Electron Corporation, Waltham, MA, USA) was used for reading the absorbance at 450 nm. All experiments were performed in triplicates. Blank PC-14/B cells and cells infected with Lv-control vectors were used as negative controls.

### Cell invasion assay

Cell invasion ability was determined using a QCMTM 24-well Fluorimetric Cell Invasion Assay kit (Chemicon, Temecula, CA, USA). The insert polycarbonate membrane was coated with a thin layer of ECMatrixTM that occluded the pores (8 μm). RPMI1640 medium (500 μL) supplemented with 10% FBS was filled into the lower chamber. After 72 h incubation, invaded cells were fixed using paraformaldehyde (4%) and stained by diaminophenylindane. Each experiment was conducted with three duplicates.

### Wound-healing assay

Cell migration ability was determined using the wound-healing assay. In brief, 1×10^5^ cells/well were seeded in six-well plates and maintained until the formation of confluent monolayer cells. Then, a 200 μL pipette tip (Axygen, Corning, NY, USA) was used for the scratching. Cells were incubated under normal conditions (37°C, 5% CO_2_) with fresh serum-free medium for 48 h. Photographs were taken using a computer-assisted microscope (Nikon, Japan) at 24 and 48 h post-scratching. Each experiment was conducted with three duplicates.

### Western blot assay

Total cellular protein was extracted from PC-14/B cells at 72 h post-infection using lysis buffer (Beyotime Institute of Biotechnology, Shanghai, China), followed by the Western blotting analysis of SIRT1, P53, and KAI1(CD82), as well as matrix metalloproteinase (MMP)-9, E-cadherin, and β-catenin using the standard methods. In brief, protein samples were separated by 10% sodium dodecyl sulfate-polyacrylamide gel electrophoresis and electrotransferred onto polyvinylidene fluoride membranes (Millipore). Primary incubation was performed using specific antibodies (1:200-600) at 4°C for 12 h. All antibodies were purchased from Cell Signaling Technology (Danvers, MA, USA). Glyceraldehyde 3-phosphate dehydrogenase (GAPDH; 1:1200, Boster Biotechnology, Wuhan, China) was used as the reference protein. Horseradish peroxidase-conjugated goat anti-rabbit/rat immunoglobulin G secondary antibodies (1:20,000, Boster Biotechnology) were used for secondary incubation. Image-Pro Plus 6.0 software (Media Cybernetics Inc., Bethesda, MD, USA) was used for the digital analysis.

### Real-time PCR measurement

Total RNA was extracted using the Trizol reagent (Invitrogen), followed by the synthesis of cDNA using the Moloney murine leukemia virus reverse transcription kit (TaKaRa, Dalian, China) with specific primers: 5’-GTCGTATCCAGTGCGTGTCGTGGAGTCGGCAATTGCACTGGATACGACTCCAA -3’ (hsa-miR-217) and 5’-TACCTTGCGAAGTGCTTAAAC-3’ (U6 snRNA [NM_001101.3]), respectively. The amplification of the miR-217 and U6 (control gene) was performed on a PCR Thermal Cycler Dice Real-Time System using the SYBR®PrimeScript PCR Kit (TaKaRa) and the amplification primers listed in Table 1. For the examination of the relative expression of SIRT1, P53, and KAI1 mRNAs, total RNA was reversely transcribed into cDNA with OligodT (TaKaRa). PCR amplification was performed using the PCR primers in Table 1. The expression of miRNA and mRNAs was analyzed with the 2-△△CT method, and all values were normalized to an endogenous U6 or GAPDH control.

### Statistical analysis

SPSS statistical software (16.0 for Windows) and GraphPad Prism 6.0 software were employed for statistical analyses. All data were expressed as means ± standard deviation (Supplementary Table 1). The difference was analyzed using a two-tailed *t*-test (between two groups) and one-way analysis of variance (among more than three groups) followed by the Tukey test. Differences with p<0.05 were considered as statistically significant.

## RESULTS

### Expression SIRT1, P53, and KAI1 in PC-14/B cells

**Figure 1:**
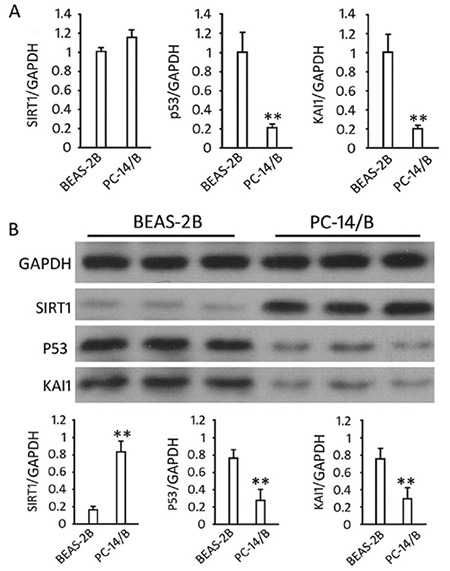
Expression of sirtuin 1 (SIRT1), P53, and KAI1 in PC-14/B and BEAS-2B cells. (A) The mRNA level of SIRT1, P53, and KAI1 in PC-14/B and BEAS-2B cells by quantitative polymerase chain reaction. (B) The protein levels of SIRT1, P53, and KAI1 in the two cells by Western blotting. Upper: representative blots. Lower: the fold change of the optical density of the target bands. Data are expressed as the mean ± standard deviation of at least three independent experiments. **P<0.01 vs controls GAPDH: glyceraldehyde 3-phosphate dehydrogenase

Compared with BEA2-2B cells, PC-14/B cells had lower mRNA levels of P53 (p=0.0039) and KAI1 (p=0.0034) and insignificant higher SIRT1 mRNA level (p=0.196; Figure 1A). Western blotting revealed that PC-14/B cells had lower levels of P53 (p=0.0007) and KAI1 protein (p=0.0007) and higher level of SIRT1 protein (p=0.0003) than BEA2-2B (Figure 1B). All these data suggested the abnormal expression of SIRT1, P53, and KAI1 in PC-14/B cells.

### Prediction and validation of the hsa-miRNA-217-SIRT1 pair

**Figure 2:**
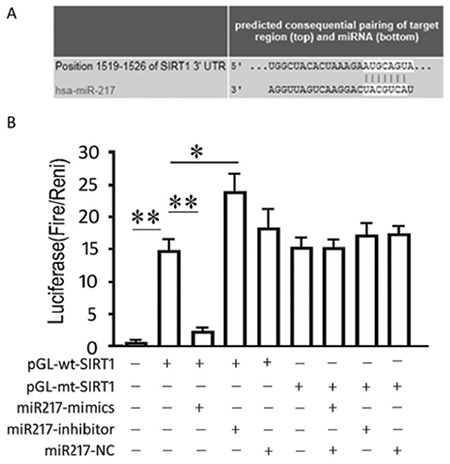
Verification of interaction between hsa-miR-217 and sirtuin 1 (SIRT1). (A) Predicted binding site of hsa-miR-217 in the 3′UTR region of the SIRT1 gene. (B) 293 T-cells transfected with pGL3-WT-SIRT1 or pGL3-MT-SIRT1 lentivirus and with or without miR-217 mimic or miR-217-inhibitor. The histogram indicates relative firefly luciferase activities. Error bars represent standard deviation and were obtained from at least three independent experiments. *P<0.05, **P<0.01

TargetScan 6.1 prediction showed there was an hsa-miR-217 target region (seeding area, 5’-AUGCAGUA-3’; Figure 2A) in the SIRT1 3’-UTR region. Luciferase reporter assays showed that the addition of hsa-miR-217 mimics into 293 T-cells carrying pGL-WT-SIRT1 vectors significantly reduced the luciferase light intensity (fire/Reni; p=0.0007; Figure 2B) but not the cells carrying the mutant 3›-UTR of SIRT1 (pGL-MT-SIRT1 vectors). The addition of the hsa-miR-217 inhibitor enhanced the luciferase light intensity by contrast (p=0.0114; Figure 2B). These results suggested the direct target relationship between hsa-miR-217 and SIRT1.

### Lentiviral infection of PC-14/B cells

**Figure 3:**
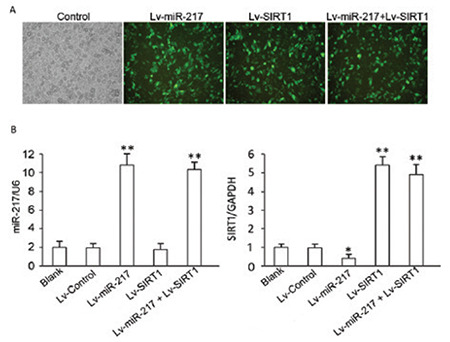
Overexpression of hsa-miRNA-217 and sirtuin 1 (SIRT1) in PC-14/B. (A) PC-14/B cells were infected with lentivirus and subjected to fluorescence microscopy to observe green fluorescent protein. (B) Quantitative polymerase chain reaction analysis of the relative expression level of hsa-miR-217 and SIRT1 at 72 h post-infection. Data are expressed as mean ± standard deviation of at least three independent experiments. **P<0.01 vs controls

The expression of green fluorescent protein in PC-14/B cells at 72 h post-infection (Lv-miRNA-217 and/or Lv-SIRT1, MOI=10) was observed under the inverted fluorescent microscope (Figure 3A). As shown in Figure 3B, Lv-SIRT1 infection increased SIRT1 mRNA by 5.28±0.33 folds (p<0.0001) and Lv-miR-217 increased miR-217 by 8.43±1.12 folds (p=0.001) at 72 h after infections. The levels of miR-217 and SIRT1 mRNA in the cells co-infected with Lv-SIRT1 and Lv-miR-217 lentiviruses were as high as those in the cells infected with Lv-miRNA-217 or Lv-SIRT1 alone (p>0.05).

### Effect of SIRT1 and hsa-miR-217 expression on invasion, proliferation, and migration in PC-14/B cells

**Figure 4:**
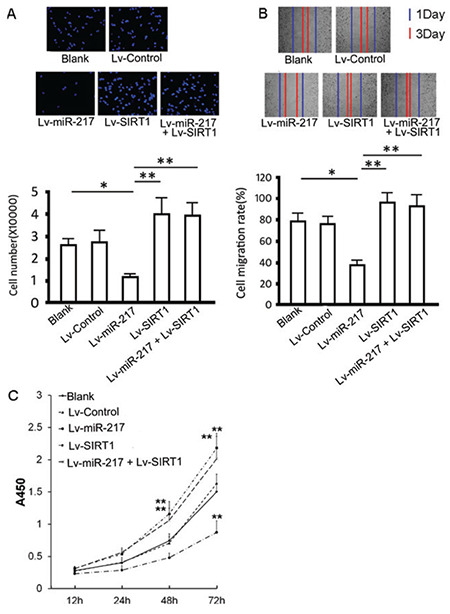
Effect of hsa-miRNA-217 and sirtuin 1 (SIRT1) expression on PC-14/B cell invasion, proliferation, and migration. Cells were infected with lentiviruses for another 72 h after identification of infection. (A) cell invasion assay. Upper: invaded cells were stained by 4′,6-diamidino-2-phenylindole. Lower: the statistical analysis of invaded cell numbers. (B) Wound-healing assay. Upper: scratch wide at 24 and 72 h post-infection. Lower: the statistical analysis of migrated cell numbers. (C) Cell viability assay. *P<0.05, **P<0.01 vs controls Lv: lentivirus

The overexpression of hsa-miR-217 significantly reduced the invaded PC-14/B cell number (p=0.004) and cell migration rate (p=0.001) compared with controls (Figure 4A and B). The overexpression of exogenous SIRT1 increased the invaded cell number (p=0.027) in PC-14/B cells compared with controls. Also, the addition of Lv-SIRT1 and Lv-miR-217 reversed hsa-miR-217-induced suppression on cell invasion and migration (p<0.05; Figure 4A and B). Cell proliferation assay showed that hsa-miR-217 overexpression significantly inhibited the proliferation of PC-14/B cells at 48 and 72 h (p<0.05 vs control and Lv-control). By contrast, the overexpression of exogenous SIRT1 increased PC-14/B cell proliferation (p<0.05 vs control) and rescued PC-14/B cells from hsa-miR-217-mediated inhibition in cell proliferation (p=0.005 and 0.006 at 48 and 72 h, respectively; Figure 4C). All these findings suggested that hsa-miR-217 inhibited the proliferation, invasion, and migration in PC-14/B cells via inhibiting SIRT1.

### Effect of hsa-miR-217 on the P53/KAI1 pathway and the expression of relevant proteins

**Figure 5:**
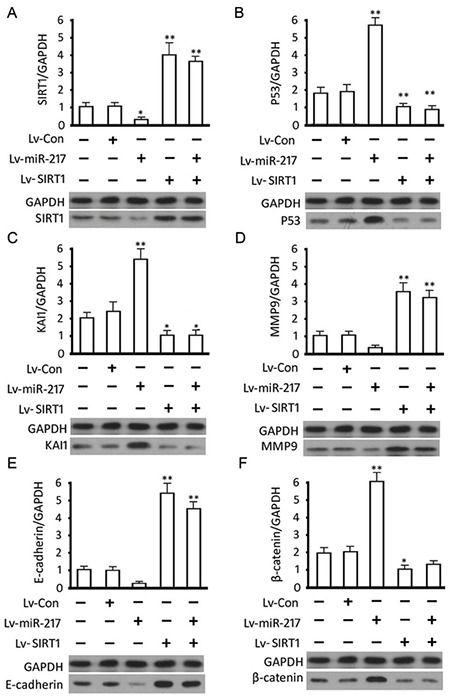
Expression of the pathway-related proteins. The fold change of (A) sirtuin 1 (SIRT1), (B) P53, (C) KAI1, (D) MMP-9, (E) E-cadherin, and (F) β-catenin protein in transfected cells. *P<0.05. **P<0.01 vs controls, GAPDH: glyceraldehyde 3-phosphate dehydrogenase Lv: lentivirus

Western blot analysis showed that the expression of hsa-miR-217 inhibited SIRT1 expression in PC-14/B cells (p=0.0.50 vs control) and that Lv-SIRT1 infection increased SIRT1 protein level (p<0.0001 vs control). There was no difference in the SIRT1 level between cells transfected with Lv-SIRT1 and Lv-miRNA-217 + Lv-SIRT1 (p>0.05) due to the lack of the wild 3’UTR in exogenous SIRT1 and the loss target of hsa-miR-217. The overexpression of hsa-miR-217 increased P53 (p<0.0001) and KAI1 protein level (p<0.0001) in PC-14/B cells compared with controls, and the infection of Lv-SIRT1 decreased the expression of P53 (p=0.006) and KAI1 proteins significantly (p=0.050 vs control; Figure 5B and C). No difference was observed in the expression level of P53 and KAI1 between Lv-SIRT1 and Lv-miR-217 + Lv-SIRT1 cells (p>0.05; Figure 5B and C).

In addition, hsa-miR-217 overexpression suppressed the expression of MMP-9 (p=0.091) and E-cadherin protein (p=0.074 vs control; Figure 5D and E) but upregulated β-catenin in PC-14/B cells (p<0.001 vs controls; Figure 5F). The addition of the Lv-SIRT1, however, increased MMP-9 (p<0.0001) and E-cadherin (p<0.0001) expressions and decreased β-catenin protein expression (p=0.016) significantly (Figure 5D-F). These results suggested that hsa-miR-217 expression promoted PC-14/B cells epithelial-mesenchymal transition (EMT) via the miR-217/SIRT1-mediated P53/KAI1 and MMP-9 signaling pathways.

## DISCUSSION

Great expectations have been shown in oncology for the utility of miRNA as cancer biomarkers and therapeutic targets (18). To date, few studies are reported to describe the expression and function of miRNAs targeting SIRT1 in brain metastases from NSCLC. In the present study, we primarily focused on the interaction of hsa-miRNA-217/SIRT1 pair and its function in the metastasis of NSCLC. Hsa-miR-217 functioned as a tumor suppressor gene in several human cancers via regulating its targets like EZH2 and E2F3 (19). Our present study suggested that the expression of hsa-miR-217 showed an inhibitory effect on the migration, invasion, and proliferation in PC-14/B cells via targeting SIRT1, suggesting the potential roles of hsa-miR-217/SIRT1 signaling in brain metastasis from NSCLC.

SIRT1 is a NAD+ dependent histone deacetylase that belongs to the sirtuin family (20). Sirtuin family members (SIRT1 to SIRT7) are wildly expressed, located at different parts in the cell, and are involved in cell proliferation, inflammation, and metabolism (20). Among the sirtuin family members, SIRT1 has the longest amino acid sequence and is the best studied. Recent studies suggest that SIRT1 could both promote and inhibit tumorigenesis and is closely related to NSCLC (20,21). Lin and Peng (22) found that SIRT1 is expressed during the NSCLC progress, especially in patients with squamous cell carcinomas, and might serve as a prognostic indicator for NSCLC. Shin et al. (23) demonstrated that hypoxic inactivation of the SIRT1/5’ adenosine monophosphate-activated protein kinase (AMPK) pathway led to cisplatin and doxorubicin resistance, and a SIRT1 activator srt1720 could augment the antitumor effects of cisplatin, which was blocked by AMPK inhibitor compound C administration, suggesting the regulatory effect of SIRT1/AMPK on drug resistance in lung cancer under hypoxia. In addition, Gong et al. (20) indicated that the combination of SIRT1/2 could predict the survival of NSCLC patients. They also detected that high SIRT1 correlated with shorter recurrence-free survival (RFS) time, whereas high SIRT2/3 and SIRT5-7 expressions were associated with longer RFS time. We confirmed the overexpression of SIRT1 served as a tumor promoter gene by promoting the invasion, migration, and proliferation of PC-14/B cells but inhibited EMT. Inconsistent with our study, Han et al. (12) found that the SIRT1 expression was in positive regulation of NSCLC cell migration,and Li et al. (24) indicated that SIRT1 protected NSLCL against osteopontin-induced EMT by inhibiting NF-κB pathway activation. Han et al. (12) detected that the knockout of SIRT1 effectively inhibited the migration of A549 cells and that SIRT1 was highly expressed in brain metastasis tissues of NSCLC. These results revealed the crucial roles of SIRT1 in brain metastasis from NSCLC by acting as an oncogene.

SIRT1 is a NAD-dependent deacetylase, and P53 is a physiological substrate of it (13). The roles of SIRT1-inhibited P53 signaling have been identified in various diseases, including osteoarthritis, liver ischemia-reperfusion injury, and cancers (13,14,16). Pan et al. (25) revealed that the expression of nicotinamide phosphoribosyltransferase (NAMPT) was a poor prognostic marker for patients with colon cancer. The increased NAMPT expression was found in subjects with rectal localized colorectal cancer compared with that in colon localized cancer. Mechanistically, the NAMPT suppression induced by colon cancer cell proliferation inhibition was mediated by repressing SIRT1 and cyclin D1/E1/E2/E3 and upregulating P53, p21, and Caspase-3 (25). Similarly, Ye et al. (16) reported that the miR-34a expression inhibited human esophageal squamous cancer cells growth via inhibiting SIRT1 and upregulating P53 and p21. KAI1 is a metastasis suppressor gene and a direct target of tumor suppressor P53 (26,27). Our study demonstrated that the expression of SIRT1 inhibited P53 and KAI1. These studies suggested that the SIRT1 inhibition activated P53/KAI1 signaling that was crucial for the growth and metastasis inhibition of cancer cells.

KAI1 plays crucial roles in suppressing metastasis via regulating various mechanisms, including cell motility, proliferation, fusion, and other signaling pathways like EGFR and Wnt (17). KAI1 expression was correlated with poor survival in NSCLC patients (28). The activation of KAI1 in colorectal carcinoma inhibited the invasion and migration of cancer cells (27). Lee et al. (29) suggested that the expression of KAI1 could suppress the fibronectin adhesion-induced EMT in prostate cancer cells. An interesting result in our study was that SIRT1-mediated metastasis was accompanied by two reverse facts: MMP-9 activation together with EMT and the β-catenin signaling suppression. EMT, which could be activated by MMP-9 and Wnt, is crucial for cancer cell invasion and metastasis (30,31). However, the results in our study suggested the hsa-miRNA-217/SIRT1/P53/KAI1-mediated EMT in PC-14/B cells was modulated by β-catenin signaling pathway instead of MMP-9 axis. The activated MMP-9 in PC-14/B cells might contribute to the increased cell invasion, migration, and metastasis.

Our study suggested the hsa-miR-217 and SIRT1 played as metastasis suppressors and promoters in NSCLC, respectively. SIRT1-mediated metastasis was related to the suppression of P53/KAI1 and β-catenin signaling. The hsa-miR-217/SIRT1/P53/KAI1 axis played crucial roles in PC-14/B cell metastasis. Our study provided a novel insight into the role of the hsa-miR-217/SIRT1 axis in human brain metastases from NSCLC. This axis might be a promising molecular target for therapy of brain metastases from NSCLC.

## Figures and Tables

**Table 1:**
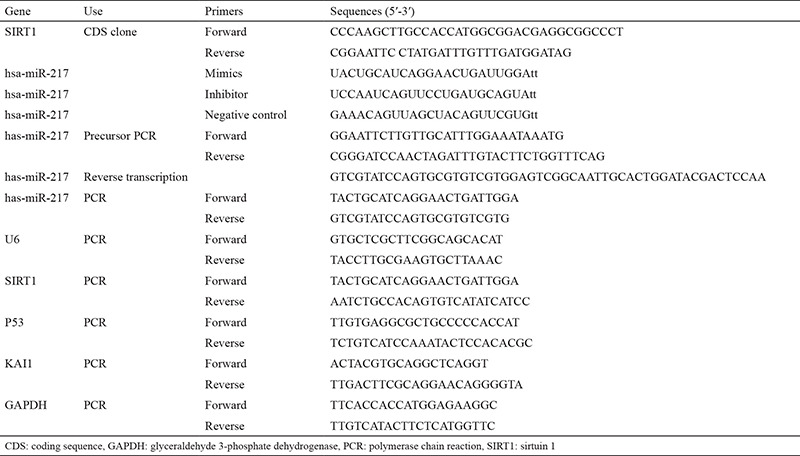
PCR primers sequences used in this study
